# The Writings of Hippocrates and Galen

**Published:** 1846-11

**Authors:** 


Art. IX.—*The Writings of Hippocrates and Galen.* Epitomised from the original Latin Translations. By John Redman Coxe, M.D., Member of the Batavian Society of Sciences at Haarlem; of the Royal Medical Society of Copenhagen, &c. *Multa re nascentur.* Philadelphia: Lindsay & Blakiston. 1846. 8vo. pp. 681.

This is far too interesting a production to be dismissed with a mere announcement of its appearance, and that at present, is all that we can give. We shall avail ourselves of the first opportunity to return to it, when we shall be able to satisfy our readers that it is a volume of rare interest. The thanks of the profession are due to Dr. Coxe for having made accessible to it a portion of the writings of the founders of medical science.

